# People at Risk of, or with Cardiovascular Diseases’ Perspectives and Perceptions of Physiotherapist-Led Health Promotion in Cameroon: A Mixed-Methods Study

**DOI:** 10.3390/ijerph21101386

**Published:** 2024-10-19

**Authors:** Etienne Ngeh Ngeh, Sionnadh McLean, Christopher Kuaban, Rachel Young, Ben W. Strafford, Joanne Lidster

**Affiliations:** 1 Research Organization for Health Education and Rehabilitation-Cameroon (ROHER-CAM), Mankon, Bamenda P.O. Box 818, Cameroon; ckuaban@yahoo.fr; 2 Department of Allied Health Professions, Sheffield Hallam University, L108, 36 Collegiate Crescent, Sheffield S10 2BP, UK; r.young@shu.ac.uk (R.Y.); j.lidster@shu.ac.uk (J.L.); 3 School of Allied Health Sciences, Charles Darwin University, Darwin, NT 0810, Australia; sionnadh.mclean@cdu.edu.au; 4 Faculty of Medicine and Biomedical Sciences, University of Yaounde I, Yaounde P.O. Box 4021, Cameroon; 5 School of Sport and Physical Activity, Collegiate Hall, Collegiate Crescent, Sheffield S10 2BP, UK

**Keywords:** cardiovascular diseases, risk factors, physiotherapist-led health promotion, Cameroon

## Abstract

Cardiovascular diseases (CVDs) and their risk factors are a major cause of illness and death worldwide, especially in low- and middle-income countries like Cameroon. Physiotherapist-led health promotion (PLHP) has proven effective in improving health and reducing CVD risks. Understanding patient perspectives is crucial for designing effective, context-specific PLHP interventions. This study explored patients’ views, experiences, perceived usefulness, acceptability, and preferred methods of PLHP, through a sequential explanatory mixed-methods approach. The quantitative data highlights a significant burden of CVD conditions and risk factors among patients seen in physiotherapy services. Qualitatively, three themes were identified and included: (1) perspectives and experiences of people at risk or with CVDs (pwCVDs) on PLHP; (2) perceived usefulness and acceptability of PLHP; (3) preferred delivery methods of PLHP. Participants reported positive feedback on PLHP and physiotherapy services. Barriers to effective PLHP included high workloads for physiotherapists, limited service access in rural areas, and prohibitive costs. Despite these challenges, participants expressed strong confidence in physiotherapists’ competence, though they also called for improved regulation and ongoing professional development. PLHP components, especially physical treatment and dietary advice, were deemed highly useful and acceptable. Patients suggested various delivery methods, including peer support groups, home visits, and mass media interventions. This study highlights the need to improve the scope of practice, competence of physiotherapists, and accessibility of physiotherapy services in Cameroon for pwCVDs. It is necessary to adopt multidisciplinary approaches to achieve better outcomes for risk factors like diabetes and hypertension in context.

## 1. Introduction

Cardiovascular disease (CVD) and associated risk factors are responsible for a significant proportion of morbidity and mortality burden of all non-communicable diseases (NCDs) globally [1]. The global prevalence of CVD increased from 271 million in 1990 to 523 million in 2019, with an associated increase in deaths from 12.1 million to 18.6 million over the same period [2]. The majority of all CVD mortality occurs in people under 70 years of age, with low- and medium-income countries (LMIC) accounting for over 85% of these deaths [2]. This suggests that effective, context-specific interventions are still required to reduce this burden in LMIC [3].

In many LMICs, health systems are highly strained due to the double burden of infectious diseases and rising incidence of NCDs [4]. CVD accounts for approximately 10% of all hospital admissions in Sub-Saharan Africa [5]. One African study demonstrated an increase in the trend of CVD admission from 4.6% to 8.2% over a decade (2004–2015), representing a 78% increase over this period [6]. The increasing burden is associated with concurrent increases of modifiable risk factors such as tobacco use, unhealthy diet, overweight and obesity, physical inactivity, and harmful use of alcohol in LMIC like Cameroon [7].

Cameroon, located in Central Africa, has an estimated population of over 29 million inhabitants as of 2024 [8,9]. Cameroon’s decentralized health system operates at three levels: central, intermediate, and peripheral, with the Ministry of Public Health overseeing the management and implementation of public health services [10]. Approximately 6.4% of the population is covered by health insurance, and the burden of healthcare is on households, with 64% of the households being unable to access healthcare due to high costs [11].

The burden of NCDs in Cameroon is rising, with risk factors overtaking some regional and global prevalence statistics. For example, 30.8% of Cameroonian women were living with high blood pressure in 2015 compared to 20.1% of women globally [12]. The prevalence of diabetes in Cameroon is estimated at 6% higher than in Africa at 3.85% prevalence [13,14]. Approximately 43.8% of Cameroonians use tobacco, compared with global figures of 36.1% [12]. An estimated 26% of Cameroonians live with hypercholesteremia compared to 25.5% among adults in Africa [15,16]. Hypertension is responsible for 41.3–54.49% of heart diseases in Cameroon [17,18]. CVD accounted for 10–16% of all hospitalizations in Cameroon, with the most prevalent CVDs being heart failure (38.5%), stroke (33.3%), and uncontrolled hypertension (22.4%) [5].

Healthcare providers in Cameroon need to address the challenge of avoidable risk factors to decrease the prevalence of CVD. There are limited effective health promotion (HP) strategies and implementation programs, and this, coupled with an undeveloped and fragile health system, is contributing to the rising risk factors and CVD [5,19]. There is increasing evidence that public health interventions are cost-effective and that secondary prevention models such as cardiac rehabilitation (CR) provide several benefits to patients [20,21]. Despite these benefits, promotive and preventive intervention programs such as HP and CR uptake and adherence in resource-limited settings like Cameroon remain poor. This is due to various factors, including a lack of trained health personnel, limited resources, competing priorities, affordability, accessibility issues, and lack of insurance coverage [22]. While there are extensive HP and prevention programs for people at risk or with CVD (pwCVDs) in high-income countries (HICs), they are either limited or completely absent in low-resource settings such as Cameroon [21].

With the absence of established HP and prevention programs in Cameroon, physiotherapists, with their core knowledge of physical interventions, exercise, and health education, are well placed to assume this role [23,24]. Physiotherapist-led health promotion (PLHP) may be a viable way to introduce or augment promotive and preventive interventions for pwCVDs. Physiotherapists manage a wide range of these patients and can engage them in HP and disease prevention through brief interventions (opportunistic advice, discussion, negotiation, or encouragement on various key public health issues) [25,26]. Enabling meaningful patient involvement results in better-informed decisions regarding care choices and aligns patient preferences with the resources at hand, which is crucial for successful lifestyle interventions [27]. This can improve understanding of health issues, context, surrounding circumstances, relevant health needs, personal values, preferences, and concerns about the proposed course of action [27]. As patient engagement becomes more effective, it fosters shared decision-making among practitioners and patients, thereby enhancing patient’s long-term adherence to interventions. [28,29]. Patients’ perceptions of healthcare providers significantly impact their adoption and adherence to lifestyle changes [30,31]. Having real patients articulate their fears and concerns about PLHP may allow physiotherapists to appreciate their patients’ perspectives, build trust, and provide care and education with better outcomes [27].

There is a lack of data on patient preferences and acceptability of HP interventions [32]. Closing this evidence gap is crucial for delivering quality, compassionate, and safe care, particularly in Cameroon, with its rising prevalence and burden of pwCVDs. A mixed-methods study is suitable for this topic because it integrates the strengths of both qualitative and quantitative research methodologies to increase understanding and provide more comprehensive answers to research questions [33]. Thus, the aim of this mixed-methods study was to explore the perspectives of Cameroonian pwCVDs’ experiences, needs, and preferences regarding a PLHP intervention and its delivery.

## 2. Materials and Methods

### 2.1. Study Design and Theoretical Underpinning

This study employed a two-part, sequential explanatory mixed-methods design following guidelines and recommendations for conducting and reporting mixed-methods studies [22,33]. This mixed-methods study was underpinned by a pragmatic paradigm and conducted in two phases [33]. In the first phase, a descriptive, cross-sectional survey was conducted to assess pwCVD views on PLHP in Cameroon. The specific objectives were: (i) to investigate the experiences of pwCVDs regarding their health and PLHP, and (ii) to establish which components of PLHP pwCVDs they might find useful and acceptable. Data was collected from 2 October 2023 to 29 December 2023. In the second phase, semi-structured interviews were conducted with a purposive sample of the surveyed participants to develop an in-depth understanding of their views of PLHP in Cameroon from 15 January 2024 to 16 February 2024. The specific objectives of the qualitative study were (i) to explore the experiences and concerns of pwCVDs on PLHP, (ii) to gain an in-depth understanding of which components pwCVDs find useful and acceptable, and (iii) to explore the preferred methods of PLHP delivery among pwCVDs

### 2.2. Study Setting

Physiotherapy practice in Cameroon lacks specialist physiotherapy services in either the public or private sectors [34]. Patients access physiotherapy services directly from home (self-referral), through referrals by other healthcare providers in hospital settings, or through referrals from healthcare institutions without physiotherapy services. A small number of physiotherapists also work in private practice. The majority of physiotherapists are concentrated in urban areas, with very little formal provision of physiotherapy services in rural areas [35]. Therefore, patients in many rural areas who need physiotherapy services may need to travel to urban areas to access these services in established clinics and hospitals.

The study was conducted in physiotherapy services across four cities in Cameroon, namely, Bamenda, Buea, Douala, and Yaoundé, to enhance access to an optimal number and diversity of pwCVDs. The physiotherapy services of the following hospitals were used: Regional Hospital, Bamenda; Military Regional Hospital, Yaoundé; Laquintinie Hospital, Douala; Buea Regional Hospital; IDIMED Polyclinic, Douala; Yaoundé General Hospital; the National Centre for the Rehabilitation of Persons with Disabilities, Yaoundé; the University Teaching Hospital, Bamenda Regional Hospital, and Limbe Regional Hospital.

### 2.3. Study Population

All participants were pwCVDs accessing physiotherapy services for the management of their condition and other related health concerns or complications. To be included in the study, participants had to be eighteen years or older, present with at least one risk factor for CVD (hypertension, diabetes, overweight and obesity, dyslipidemia), or present with a diagnosis of CVDs, which may include heart failure, surgical heart condition, coronary artery disease, coronary heart disease, stroke, or myocardial infarction [7]. All participants received physiotherapy for at least two weeks and could communicate well in French or English. Exclusion criteria included people with long-term cognitive or communication impairments preventing them from providing informed consent, for example, people living with Alzheimer’s disease and dysphasia. People who live with pre-existing psychotic illness, such as schizophrenia, or those receiving end-of-life care were also not eligible to take part in the study.

### 2.4. Recruitment

Practicing physiotherapists in participating services identified all eligible participants. Trained data collectors, i.e., physiotherapists, students on placement or the investigator (EN), then contacted the potential participant to explain the objectives of the study and procedure. A screening form with the inclusion and exclusion criteria for the study was used to assess participant eligibility. Each eligible participant, depending on his/her preference, was given an information sheet in English or French for the study. When consent was granted, the survey instrument was then administered electronically (Phase 1). During this phase, all consenting participants were asked whether they would be willing to take part in a follow-up interview (Phase 2). Participants provided their preferred contact details for later use in arranging the interview.

### 2.5. Phase 1: Quantitative

#### 2.5.1. Patient Survey

The survey instrument was designed to collect the following data (Supplementary File S1): Section A; demographic information, Section B; perception of pwCVDs towards PLHP, and Section C; acceptability and usefulness of PLHP. The survey took approximately 10–15 min to complete. The completed survey instrument was piloted, and all feedback was used to improve on the survey instrument before final administration.

#### 2.5.2. Training of Data Collectors

Data collectors were trained by the lead author (ENN) in the selected hospital establishments. This consisted of two physiotherapists and three physiotherapy students on clinical placement to enhance the continuity of data collection in the absence of the principal investigator. The training was directed toward understanding the purpose of the study, how to deliver the information sheet and seek consent, and how to access the survey tool, complete it, and submit it electronically.

#### 2.5.3. Sample Size Calculations

Calculator.net was used to estimate the sample size for the survey [36]. We used an estimated annual population size of 2000 pwCVDs across the participating hospitals and a population proportion of 90% with an eligible condition. With a significance level set at 0.05 and a margin of error of 5%, the sample size for this study was estimated to be approximately 130 participants. Other similar patient surveys examining patient preferences for treatment of LBP, inflammatory bowel disease, spinal surgery, and diabetes have recruited 130–170 participants [37,38,39]. To consider errors within estimates, the target sample was set at an upper limit of 170 participants.

#### 2.5.4. Data Collection

The survey instrument was administered electronically via a link using mobile phones, tablets, iPads, and laptops, independently by the patient or with the aid of a data collector. Where participants consented but had no means to complete the survey, the investigator or trained data collector facilitated completion on electronic devices dedicated to the study.

#### 2.5.5. Data Analysis

Data were checked in Qualtrics. The data were then downloaded in Microsoft Excel 365 format and transferred to SPSS (IBM SPSS Statistical Software, version 26.0) for statistical analysis. Descriptive statistics (frequencies, central tendency, dispersion/variation, and percentages) were used to present the participants’ demographic information and pattern of practice. All data were considered in the analysis regardless of missing responses.

### 2.6. Phase 2: Qualitative Interviews

#### 2.6.1. Sampling and Recruitment

Participants were selected purposively from those who provided preliminary consent to follow-up interviews as part of completing the survey (phase 1). This was based on gender, level of education, duration of receiving physiotherapy, various characteristics typical of pwCVDs, and geographical locations of the patients. All participants were contacted via their preferred contact details, which they provided in Phase 1. Recruitment was carried out through telephone and in-person communication. Once participants provided written consent, arrangements were made to conduct the interview in a mutually convenient place, considering the participant and investigator’s safety. For consistency, before each interview started, the investigator read a script that explained the purpose of the study, that participation was voluntary, that there were no known risks for participation, and what to expect in terms of content and duration of the interview.

#### 2.6.2. Data Collection

Interviews were conducted face-to-face using individual semi-structured questionnaires. Permission was obtained from each participant to record the interview to ensure the accuracy of the resulting transcripts [40]. A topic guide (Supplementary File S2), informed by previous literature, facilitated the interviews [41]. The investigator used a general warm-up question specific to the patient’s condition before addressing all central questions to build rapport in the presence of the audio recording device [42]. The interview recording was transcribed verbatim, including verbal expressions and body language that revealed considerable emotions and or expressions of feeling on specific issues [43]. A field diary was kept with comments, explanations, descriptions, and interpretations of patients’ responses.

#### 2.6.3. Sample Size Determination

While a range of 3 to 10 participants is considered appropriate for qualitative studies using semi-structured interviews [44], a similar physiotherapy qualitative study used 13 participants [45]. We aimed for an upper limit of 20 participants as recommended for studies of lived experience or without a well-defined cultural domain [46]. This upper limit was assumed to provide adequate information based on the aims and the underdeveloped nature of the PLHP for pwCVDs [47]. However, data collection was stopped at 13 interviews as data saturation occurred, and no further additional themes emerged from the dataset [48].

#### 2.6.4. Data Analysis

The qualitative data collected was analyzed using a two-stage reflective thematic analysis [49,50]. The first stage employed the 6-step thematic approach recommended by Braun and Clarke [49], and the second stage was a methodological reflection to understand and derive meaning from the generated data in context [50]. The interviews were transcribed and coded using NVivo 12 [51]. The reviewers (E.N.N. and B.S.) used the results of the first interviews to develop common categories and subcategories, which were then clarified and supported with quotes from an interview transcript. The general categorization evolved until the end of the analysis [49].

The methodological reflection was based on the categories obtained from the descriptive analysis, and conceptual categories were developed beyond merely describing the themes and subthemes to give interpretation to the thematic descriptions that emerged from the interviews [50].

Two researchers (E.N.N. and B.S.) conducted a joint analysis after they had analyzed each interview separately. Interviews took between 21 min and 38 min. We concluded that data saturation had been achieved by comparing interview data throughout the analysis process and observing that no new aspects, dimensions, or nuances of codes were emerging. The interpretation of the interviews was verified by two co-investigators (R.Y. and S.M.). The findings were written according to guidelines for reporting qualitative research [52].

### 2.7. Reflexivity

Engaging in reflexive thematic analysis requires researchers to acknowledge and express the underlying assumptions guiding their approach and interpretation of qualitative data [53]. In this study, coders practiced reflexivity during the coding process by considering their professional role and research biases that could impact the interpretation of qualitative responses. The two qualitative coders in this study (E.N.N. and B.S.) identify as black and white males, emerging and early middle-aged adults, with an interest in health promotion. Throughout the coding process, they remained mindful of their biases and endeavored to assign themes based on data rather than their own biases informed by previous research and knowledge [53].

### 2.8. Triangulation and Integration of Findings

This study adopted and reported triangulation of data during data analysis and interpretation based on the methods (survey and qualitative findings), investigators (multiple observations), and data source (pwCVDs) at the end of the study [54]. The triangulation process involved the initial sorting of themes and categories from the qualitative data and relating it to the quantitative findings to create a coding matrix. The data was then analyzed to see if there was agreement, dissonance, or partial agreement between the data sets [54].

## 3. Results

### 3.1. Participant Characteristics

A total of 146 respondents and 13 participants in Phases 1 and 2, respectively, were included in the final analysis, with the characteristics summarised in Table 1. In phase 1, most respondents were females (52.7%), with the average mean age of all participants being 46.18 ± 14.77 years. Respondents with primary education (29.0%, n = 42) and undergraduate degrees (20.7%, n = 30) constituted higher proportions for education levels. Most respondents (68.4%, n = 100) had multiple risk factors, with hypertension (60.9%, n = 89) and overweight (56.8%, n = 83) being the most prevalent. Stroke (45.2%, n = 66) was the most prevalent CVD and was associated with multiple risk factors. The most prevalent symptoms were swelling of arms and legs (38.3%, n = 56) and poor sleep (34.2%, n = 50). The greatest challenges for pwCVDs included taking part in physical activity or exercise (56.8%, n = 83), doing usual activities of daily living (56.0%, n = 80), and making hospital visits (31.0%, n = 44). Most respondents on medications (73.3%, n = 117) followed medical recommendations for health promotion or medical management of chronic conditions.

Thirteen participants completed the semi-structured interviews, including males (n = 7, 53.8%) and females (n = 6, 46.2%) with a mean age of 56.31 ± 12.11 years ranging from 37 to 79 years. Participants had established CVD and multiple risk factors. Detailed characteristics of interviewed participants can be found in Table 2.

### 3.2. Synthesised Findings

**Figure 1:**
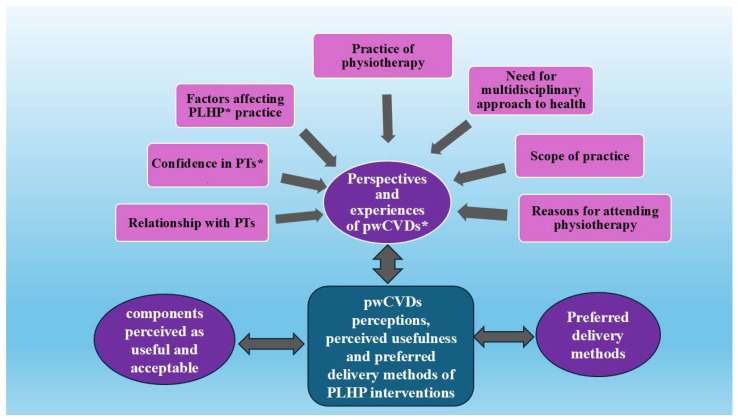
Summary of the qualitative findings showing the first and second-order themes of phase 2. * PLHP: physiotherapist-led health promotion, pwCVDs: People at risk or with cardiovascular diseases, PT: physiotherapist.

Analyses of the transcripts generated three themes, which included: (1) perspectives and experiences of pwCVDs on PLHP, (2) perceived usefulness of PLHP by pwCVDs, and (3) preferred delivery methods of PLHP (Figure 1). Complete and independent results of both the quantitative and qualitative phases can be found in Supplementary File S3.

### 3.3. Perspectives and Experiences of pwCVDs on PLHP

#### 3.3.1. Reasons for Attending Physiotherapy

The quantitative findings demonstrated a diverse range of CVDs and risk factors, with stroke as the leading CVD (93.0%, n = 66), hypertension (36.77%, n = 89), and overweight (34.30%, n = 83) as the leading CVD risk factors. The leading symptoms among respondents were swelling of the limbs (38.36%, n = 56) and poor sleep (34.25%, n = 50). The qualitative findings demonstrate that some participants access physiotherapy services primarily due to cardiovascular conditions or risk factors, while others had cardiovascular issues identified as secondary concerns, such as being overweight, having diabetes, and hypertension. This could be following traumatic incidents or low back pain, among others.

“*I started physiotherapy because of insomnia and being overweight*”.

(P3)

“*I was a victim of an accident. I was operated upon, and I was unable to move. I was bedridden. So, after the operation, the doctor advised that I could go for physiotherapy to help speed up the recovery. So, just like a week after the operation, I started physiotherapy*”.

(P7)

#### 3.3.2. Scope of PLHP Practice

The quantitative findings demonstrate that 58.9% (n = 86) of the respondents believe that the role of physiotherapists is not limited to exercise prescription. Qualitative findings also demonstrated that patients felt that although physiotherapists emphasized the importance of exercise and physical activity, they could also have a wider role in delivering other relevant aspects of HP. Most participants reported that they had received and appreciated information on a range of PH topics related to alcohol (n = 3), diet (n = 8), etc., even though PTs might not be experts in these areas. Physiotherapists less frequently and superficially covered HP activities related to sleep, lifestyle, etc. The majority of participants felt that physiotherapists could be delivering more strongly in a greater number of HP activities.

“*What they do is just exercise, but as concerns nutrition and other health issues. I think no one has ever told me about it, regarding stress management, nothing*”.

(P11)

“*I don’t really know some areas of the therapist’s scope of work, but they can advise us on diet which is good*”.

(P10)

#### 3.3.3. Relationship with Physiotherapists

The majority of the respondents indicated that their physiotherapists have never made them uncomfortable (81.4%, n = 118) and can form good relationships with patients (79.45%, n = 116). Qualitatively, most participants (12/13) reported having very healthy and supportive relationships with physiotherapists, although 1/13 participants reported that not all physiotherapists form good and healthy relationships with their patients.

“*No, there are very interesting people, ‘he laughs’ for my own sessions that I had were very interesting, very interesting. A lot of encouragement and you could see results*”.

(P3)

“*But others look a little bit, more of military kind of harsh to patients, some of them, but not all. Others are wonderful*”.

(P12)

#### 3.3.4. Physiotherapy Practice

The quantitative data shows that less than half of respondents had discussions about treatment goals and objectives (47.9%, n = 69). The qualitative findings also complemented this result. Three participants (3/13) were aware of the importance of goal setting in physiotherapy practice and acknowledged that they have yet to have discussions around treatment goals with their physiotherapist. Some participants were not aware of this, but some were aware that it is part of the approaches to physiotherapy practice.

“*The first thing I would like to know from the physiotherapist is to know about my goals, which I never had. I have a problem with my lower back. At the end of the therapy, I should be able to do what I was unable to do before starting the therapy. So, that should be my goal at the end of my treatment. If I achieve my goal, then I know that things went well. Yes!*”.

(P10)

Participants also perceived that physiotherapy is not well regulated, and the training facilities are limited. Consequently, this led to a shortage of physiotherapists in many hospitals. Participants hold the view that physiotherapists should be professional in their approach and obtain contemporary and standardized training to be effective practitioners.

“*First of all, I must say that what I’ve noticed that they are charlatan physiotherapists*”.

(P1)

“*So they really need to increase the number of people that are training in this area because you go to some hospitals, and you hardly find a physiotherapist. Then most of the time you have nursing aid assistant that have been trained informally in hospital settings, and they are handling this type of issues*”.

(P3)

#### 3.3.5. Need for a Multidisciplinary Approach to Health

The majority of respondents on medications (73.3%, n = 117) followed medical recommendations for either health promotion or medical management of chronic conditions. The swelling of arms and legs (38.3%, n = 56) and poor sleep (34.2%, n = 50) were the most prevalent symptoms amongst the respondents. Qualitatively, participants held the view that physiotherapists cannot treat and manage all conditions and risk factors, such as diabetes and hypertension, independently and should work in synergy with other relevant clinicians.

“*But in the real treatment, I think that the specialists of this area are more competent if they want to take it as a disease to treat. But for the physiotherapists, I see him handling it as something resulting from his own specialty and something which his advice can equally help to check. But for treatment per say, I don’t think so*”.

(P1)

#### 3.3.6. Factors Affecting PLHP Practice

Our quantitative data shows only 18.6% of respondents (n = 27) think that the workload of the physiotherapists limits their engagement in HP. The majority (40.6%, n = 59) think workload has no impact on physiotherapists’ engagement in HP. The qualitative data suggests that participants believe the workload for the physiotherapy service was too much and may impact the ability of the physiotherapist to deliver HP messages. This then also translated to prolonged waiting time for the patients.

“*So I just felt that they have a lot of work because the person that comes here is working elsewhere. He has full-time work. So when he comes here, maybe he has to rush to his office or has other patients to see, I just feel like they are not putting enough time*”.

(P4)

“*I think everything boils down to time. Sometimes I feel that I don’t have enough time, the workload at the level of the physiotherapist (long waiting time)*”.

(P2)

In addition, some participants (12/13) believe there is a significant demand for physiotherapy services, and people are suffering unnecessarily because they are unaware of the service’s role and importance. They expressed concerns about limited access, especially for patients in rural areas, as services are primarily available in urban areas with a limited workforce. The number of physiotherapists needs to be increased to meet the growing demand. Participants suggested that, in addition to increasing the number and quality of physiotherapists, the government should provide subsidies and insurance policies to help citizens access these services, as the cost of physiotherapy sessions was identified as a major barrier to accessing the services, limiting the number of sessions for some and rendering therapy ineffective for others.

“*Well, for the cost. I won’t say it is quite affordable because someone who has to do physiotherapy, let’s say, for 2 months, 3 months, and people go even beyond that. If you have to pay because, at the beginning, I had to come every day. So considering the standard of living here in our country, I think that the cost is not quite affordable for everyone*”.

(P7)

“*Subvention for citizens, the government chooses to subsidise this issue, if they want the improvement of their citizens, it’s not all about money the government rules citizens. It doesn’t rule people. People pay taxes because of their health care*”.

(P11)

There is a belief among some participants that the government should implement policies for public awareness and education on health issues based on national statistics, emphasizing that public health intervention should be strengthened rather than leaving health issues to individual patients.

“*You know things that come from the government media is valued by the populace, where people believe that if they’re saying this thing now and bring it out, you see the specialists coming to talk, people are taking more seriously because they are giving statistics, national statistics. At the macro level, what is happening in the nation with this and the consequences? People will take it more seriously rather than me going informally and doing my Google search and checking on it; when they start talking like that, it becomes a policy issue. Yes, and people will take it more seriously*”.

(P11)

Most respondents (66.67%, n = 96) reported that physiotherapists always praise and motivate them to reach their goals. Qualitative findings indicate that some participants (3/13) found visiting physiotherapy services more beneficial than joining sports clubs or exercising independently, as they struggled to maintain motivation on their own. They also noted that the lack of equipment and inadequate investment and training in physiotherapy services by hospital administrations contribute to the poor performance of these services.

“*So I feel better here. If they can make it more frequent per client, it will be nice. I come and exercise, somebody follows me, is very nice, in the club is not the same. People are doing it their own and, in the club, you may injure one part of your body. Just because they just do their business you know, they are not specialists most of the time*”.

(P3)

“*Alone I cannot do it but when I come here, they are very specific. And I do not really like it alone because whenever I’m tired, I just get up and go. But here they say, no, 15 min, you still have 3 min, and I make the effort*”.

(P13)

Additionally, participants (3/13) felt that some of the education and advice from physiotherapists were unclear and not patient-centered, making them difficult to follow. The advice sometimes seemed passive, as patients felt instructed to follow it without understanding its importance. Participants expressed a desire to extend PLHP services to home visits or self-delivery.

“*Yeah, because what I’m saying is that when you tell him to do some physical exercise, which by his very nature, he cannot do it, it means nothing. So, it is good to tell somebody what he can do. I’ve had the experience where I’m told what to do, which I cannot do*”.

(P1)

“*I would love that. But because I am having challenges, I think that doing it in a group might not be very comfortable working in a group. That’s why I always like home visits; I’m still striving for home visits*”.

(P5)

#### 3.3.7. Confidence in Physiotherapists

Quantitatively, 60% of respondents (n = 87) believe that physiotherapists have the skills to assist them with their conditions. This was further supported by the qualitative findings, as the majority of the participants expressed strong confidence and trust in the physiotherapy workforce. While some participants (4/13) expressed reservations about fully trusting their physiotherapist, they still found them to be very helpful in their practice. Regarding training and competence, participants acknowledged that physiotherapists are generally competent but noted there is room for improvement. Some participants (7/13) felt that, regardless of their perception of competence, the managers of physiotherapy establishments should ensure competence and standards of practice. Also, some participants believe that physiotherapists need ongoing support and supervision in their practice.

“*The physiotherapist wants me to be well. So, I don’t think that is any advice or restrictions that they can give me, which can be contrary to essence, I don’t think so. What I got, help me and for me, what they do, I don’t see anything to object*”

(P4)

“*Most of the time they are lacking, and they need somebody who should be overseeing them, who is actually very professional overseeing them, especially in the government settings*”.

(P3)

### 3.4. Perceived Usefulness and Acceptability of PLHP

#### 3.4.1. Components Found to Be Useful

The quantitative data shows that the majority of the respondents perceived PLHP to be very useful, with a mean score of 86.8% (n = 124) across all components (i.e., diet, sleep, stress management, etc.). The component with the highest proportion of perceived usefulness was dietary advice on fruits (91.6%, n = 132), and the lowest was advice or education to stop smoking (75.35%, n = 107). The qualitative data revealed that participants viewed diet and nutrition as a crucial aspect of PLHP, recognizing the importance of combining it with other components. Some participants also deemed physical treatment essential, including exercises on the treatment bed. Most participants found the education and interventions provided by physiotherapists to be universally useful, without a preference for specific components.

“*What I know is that their advice, in general, is useful. Yes, I know that is useful, but to say, I can choose one for the other, no*”.

(P1)

“*No, no, “Nodding in disagreement” they should talk about everything. They should talk of everything that will help me*”.

(P6)

#### 3.4.2. Components Found to Be More Acceptable

The majority of the respondents found PLHP advice/education acceptable across multiple components, with a mean score of 94.65% (n = 131). Increasing general physical activity advice was the component that was most acceptable (95.80%, n = 137). Conversely, advice on alcohol consumption had the highest rate of unacceptability, with 4.8% (n = 7) of respondents. This is because respondents who do not drink alcohol do not value such counsel. Qualitative findings indicated that participants viewed these interventions and educational efforts as highly beneficial and essential for managing their conditions. They emphasized that such resources should be publicly accessible, with physiotherapists playing a key role in leading exercises, planning, and educating the public, given their crucial impact on health outcomes.

“*When I suddenly had a stroke in 2013, it was necessary for me to get to physiotherapy for treatment. Well, I can say that was an important element in my treatment because they said it. Yes, I was heavily encouraged to do that. Let me add that it helped me a lot*”.

(P1)

“*Physiotherapists hold a transformational position in people’s health*”.

(P9)

### 3.5. Preferred Methods of PLHP Delivery

This aspect was only explored during the qualitative phase of the study. Participants proposed and justified a variety of delivery methods based on their experiences, conditions, circumstances, or observations of the physiotherapy service. Patient peer support groups were considered beneficial for sharing experiences and challenges to improve adherence to PH interventions. Due to personal challenges such as communication or mobility issues, some participants valued privacy and preferred home visits, although they acknowledged that arranging and maintaining these visits can be costly. Participants also advocated for one-on-one discussions or education, citing reasons such as mobility issues, different pathologies, and the need to clarify doubts. Some participants preferred group discussions and lectures with physiotherapists, as this saves time and ensures effective communication, especially for people with similar conditions. Others suggested that regular audiovisual or mass media slots could have a broader impact on disease prevention and health promotion. Some preferred workshops, seminars, or regular talks, provided they are invited in advance, either free or at a minimal cost. Participants also mentioned positive experiences with using apps and expressed interest in having access to them. Participants also highlighted that written or printed information is helpful for various reasons, though a few found it challenging due to reduced vision. A few participants did not prefer the delivery method but valued any information that could improve their health (Sample quotes can be found in Supplementary File S3, qualitative results).

## 4. Discussion

This mixed-methods study aimed to provide a comprehensive overview of patients’ experiences, perceptions, and preferences of PLHP for pwCVDs in Cameroon to inform intervention development. Our data reflects participants’ views of multiple risk factors and challenges and demonstrates positive perceptions of PLHP delivered via multiple methods. The qualitative and quantitative data were agreed well across the study elements.

Consistent with previous research from Ghana among stroke patients [55], approximately two-thirds (68.4%) of our respondents had multiple CVD risk factors. They, however, reported a higher prevalence of hypertension (85%) and obesity (58%) compared with 60.9% and 56.8% in our study, respectively. These differences could be explained by their study being conducted among stroke survivors [55]. Conversely, Mastwyk and colleagues in Australia reported a 37% and 58% prevalence of metabolic syndrome and hypertension among clients attending private practice physiotherapy with predominantly musculoskeletal conditions (94%). The differences could be attributed to their study population of musculoskeletal patients with potentially lower levels of CVD markers than in our study population on CVD [56]. The high levels of varied CVD risk factors reported in our study and in the general Cameroon population highlight the need for PLHP strategies adopting multimodal strategies to address this complex problem in Cameroon [5,6,13]. Physiotherapists are well-placed to deliver these HP interventions for pwCVD, other clinical populations, and the general population [24,57].

Consistent with previous research on patients’ experiences with physiotherapy services from Kenya and Spain [58,59], our participants reported excellent/good cordial relationships with physiotherapists and perceived PLHP interventions as useful and effective for managing their condition and improving their health. Despite positive perceptions, participants presented challenges related to the competence and scope of practice of physiotherapists, demonstrating the need for comprehensive training and skill development beyond exercise and physical activity to address their needs better. This supports the findings of Severin and colleagues that only 14.8% of physical therapists measure blood pressure and pay attention to examining and educating patients on CVD risk factors in the USA [60]. This may be because of specialist respiratory therapists in the USA, who tend to deal with patients with complex cardiac and pulmonary conditions [61]. Physiotherapists’ workloads due to a lack of staff and equipment resulting from prolonged waiting time were reported in our study. This is consistent with previous data reporting 0.92 physiotherapists per 100,000 population in Cameroon and similar across many African countries [58,62], which compares less than favorably with other countries such as South Africa and Germany with 13 and 234.4 physiotherapists per 100,000 respectively [61]. For participants with high levels of disability coupled with challenges to accessing physiotherapy services may mandate a shift towards community-based services to facilitate access. Facilitating community-based programs necessitates collaboration and empowerment of community-based workers and expert patient groups to increase access and sustainability through affordable mechanisms and provide effective health education [24,58].

Contrary to growing evidence that written prescription is more effective and motivating than oral advice for improving lifestyle changes such as physical activity [63,64], the majority of our participants expressed a preference for individual discussions and, to a lesser extent, regular planned educational sessions, group discussions, lectures, printed information, use of apps among others. This could be explained by the degree to which resources are available in Cameroon, including relevant evidence, the internet, and printers. Our participants also highlighted patient-related factors, including loss of eyesight with aging, making reading challenging. Oral advice was preferred because of the advantage of clarifying doubts following relevant questioning. This highlights and emphasizes the need for context and patient-centered considerations in clinical practice.

In summary, the findings of this study suggest that pwCVD perceives PLHP delivery across many components as useful and acceptable. Many positive and some negative patient experiences of physiotherapists delivering PH interventions were identified. Positive and negative experiences also facilitated or limited access to HP information or affected the quality of PLHP interventions. This, coupled with the high prevalence of CVDs and risk factors for CVD, creates an imperative for a more systematic and comprehensive approach to PLHP in Cameroon [65].

### 4.1. Implications for Practice, Training and Research

#### 4.1.1. Practice

All Physiotherapists should understand how risk factors influence the development and progression of CVD and be competent in delivering lifestyle education, diet, stress management, sleep hygiene interventions, and exercise and physical activity. Therefore, there is a mandate for the Cameroon Society of Physiotherapy (CASP), the Ministry of Public Health, and relevant stakeholders to organize professional development training that covers this wide range of HP activities for practicing physiotherapists. Community-based initiatives and collaboration with community-based health workers and expert patient groups may also be valuable options [66]. A variety of flexible PH delivery methods would support physiotherapists in delivering PH messages to a larger population that may improve compliance and adherence, leading to better health outcomes. Mass media and community outreach may also facilitate patient education and behavior change. Collaboration with other healthcare professionals is crucial to enhance the delivery of PLHP intervention and improve patient outcomes.

#### 4.1.2. Training

The undergraduate physiotherapy training courses must be comprehensive, covering key aspects of HP, such as relevant assessment tools on lifestyle-related behaviors, counselling skills, and behavior change strategies for specific HP components [24]. This will enable qualified physiotherapists to be confident in delivering HP interventions.

#### 4.1.3. Research

Additional research is needed to explore the long-term impacts of PLHP interventions for pwCVD management and outcomes in the Cameroonian context. Therefore, to allocate limited resources effectively, it is necessary to investigate the effectiveness of different PLHP components (e.g., dietary advice and stress management) in improving cardiovascular health. Research should focus on developing and testing patient-centered PLHP models considering individual preferences and barriers.

### 4.2. Strengths and Limitations

Using quantitative and qualitative methods provided a comprehensive understanding of pwCVDs’ perceptions and experiences in physiotherapy services. We also included a diverse sample of participants from four regions of Cameroon across several physiotherapy services, enhancing the generalizability of the findings. Qualitative interviews offered great depth and insights into patients’ personal experiences and perceptions of our findings. A joint analysis by a physiotherapist and a non-physiotherapist qualitative researcher enhances the quality of findings by ensuring they are data-driven and minimizing researcher bias.

The relatively small sample size, particularly for the qualitative component, may limit the generalizability of the findings. However, the recruitment of all participants in the qualitative phase was based on criteria that represented the sample, including gender, condition, and duration of exposure to physiotherapists. Secondly, reliance on self-reported data might have introduced bias, as participants might overestimate or underestimate their experiences and perceptions. Finally, the inclusion of physiotherapy services primarily based in urban settings may not reflect the experiences and perceptions of patients in rural settings.

## 5. Conclusions

The findings demonstrate positive perceptions and experiences of physiotherapists delivering a variety of HP information and advice in Cameroon with wide acceptance of PLHP interventions. However, pwCVDs also report unmet needs related to service provision, personalization, and tailoring of PLHP interventions. This finding resonates with prior literature on disciplinary workforce development for wider HP intervention engagement and delivery. This underscores the need to enhance physiotherapy entry-level training in Cameroon. Additionally, relevant stakeholders must develop strategies to extend the delivery of PLHP interventions beyond urban areas to rural and remote settings.

## Figures and Tables

**Table 1:** Sociodemographic and clinical characteristics of the participants enrolled in the study (n = 146).

Variable		n	%
Sex	Male Female	69 77	47.3 52.7
Mean age (years)	46.18 ± 14.77	146	100
Educational level	Primary school High school Secondary school Undergraduate degree Postgraduate degree Others (No formal education)	42 21 29 30 20 3	29.0 14.5 20.0 20.7 13.8 2.1
Cardiovascular diseases	Stroke Heart attack coronary heart disease	66 1 4	93.0 1.40 5.63
Cardiovascular disease risk factors	Hypertension Overweight Diabetes Regular alcohol consumer High Cholesterol Regular smoker	89 83 31 23 9 7	36.77 34.30 12.80 9.50 3.71 2.89
Frequency of symptoms and challenges posed by risk factors and cardiovascular diseases among participants	Swelling of limbs Poor sleep Dizziness Cough Palpitations Difficult breathing Chest pain Loss of appetite Vomiting Others	56 50 42 30 24 22 21 18 3 4	38.36 34.25 28.77 20.55 16.44 15.07 14.38 12.33 2.05 2.74
Do you have difficulty with any of the following because of the condition(s)? (n = 146)			
	No difficulty	A little difficulty	Difficulty
Taking part in exercise or physical activity (e.g going to the gym, taking a walk)	19.9% (n = 29)	23.3% (n = 34)	6.8% (n = 83)
Doing usual daily activities (e.g., cleaning, cooking)	23.8% (n = 34)	20.3% (n = 29)	56% (n = 80)
Following medications	70.6% (n = 101)	10.5% (n = 15)	18.9% (n = 27)
Making hospital visits	55.6% (n = 79)	13.4% (n = 19)	31.0% (n = 44)
	Not at all	Sometimes	Always
Do you follow medicine/drug recommendations suggested by your physician or other specialist?	8.9% (n = 13)	16.4% (n = 24)	73.3% (n = 117)

**Table 2:** Characteristics of participants interviewed for PLHP (n = 13).

Participants	Age/Years	Gender	Profession	Academic Qualification	Risk Factor(s) and/or Cardiovascular Condition
* P1	79	* M	Retired school administrator	Postgraduate degree	Hypertension, diabetes, stroke, high cholesterol, and CHD *
P2	49	* F	Agricultural engineer	Postgraduate degree	Overweight
P3	64	M	Community Development Officer	Undergraduate degree	Insomnia, hypertension, and overweight
P4	68	F	Retired Civil Servant	Undergraduate degree	Stroke, hypertension, overweight, and high cholesterol
P5	37	M	Unemployed	No formal education	Stroke and hypertension
P6	56	M	Business	High school	Hypertension, overweight, stroke, and high cholesterol
P7	39	F	Translator	Postgraduate degree	Overweight
P8	65	M	Business	High school	Overweight
P9	46	F	Teacher	Postgraduate degrees	Overweight
P10	60	F	Retired nurse	Undergraduate degree	Hypertension, diabetes, overweight, and high cholesterol
P11	46	M	Business	Diploma	Overweight
P12	63	M	Retired physical education and sports teachers	Undergraduate degree	Hypertension, diabetes, and overweight
P13	60	F	Retired civil servant	Undergraduate degrees	Overweight, diabetes, hypertension, physical inactivity, and stroke

* P: participants, M: male, F: female, CHD: coronary artery disease.

## Data Availability

The raw data supporting the conclusions of this article will be made available by the corresponding authors upon request.

## Supplementary Materials

The following supporting information can be downloaded at: https://www.mdpi.com/article/10.3390/ijerph21101386/s1, Supplementary File S1: The survey, File S2: Topic guide for the second phase, and File S3: The complete quantitative and qualitative results.
