# Supplementary material for: People at Risk of, or with Cardiovascular Diseases’ Perspectives and Perceptions of Physiotherapist-Led Health Promotion in Cameroon: A Mixed-Methods Study
Source: Int J Environ Res Public Health. 2024 Oct 19;21(10):1386. doi: 10.3390/ijerph21101386 (PMC11507622; doi:10.3390/ijerph21101386)
Supplement: Supplementary file 1 [file ijerph-21-01386-s001.zip › ijerph-3207391-supplementary.pdf]

S1: SURVEY

SECTION A – PERSONAL DATA

Select or complete the following appropriately

Gender

- ○ Male (1)
- ○ Female (2)

Age in years_____

What is your occupation_________

What is your highest level of education?

- ○ a. Primary school (1)
- ○ b. High school_ (2)
- ○ c. Secondary school (3)
- ○ d. Undergraduate degree (4)
- ○ e. Postgraduate degree (5)
- ○ f. Others (please specify) (6)

SECTION B: CONCERNS TOWARDS HEALTH

i) Which of the following best describe you? (Tick all that apply)

- ☐ a. High blood pressure (1)
- ☐ b. Diabetes (2)
- ☐ c. Overweight (3)
- ☐ d. regular smoker (4)
- ☐ e. Regular alcohol consumer (5)
- ☐ f. Stroke (6)
- ☐ g. High cholesterol (7)
- ☐ h. Heart attack (8)
- ☐ i. Coronary heart disease (9)
- ☐ k. Others (Please, specify) (11)

II) Do you have difficulty with any of the following because of the condition(s) above

	No difficulty (1)	A little difficulty (2)	Moderate difficulty (3)	Extreme difficulty (4)
Taking part in exercise or physical activity (e.g. going to the gym, taking a walk) (1)	○	○	○	○
Doing usual daily activities (e.g. cleaning, cooking) (2)	○	○	○	○
Following médications (3)	○	○	○	○
Making hospital visits (4)	○	○	○	○

III) Choose your most frequent symptom(s) among the following (choose all that apply).

- ▢ a. Difficulty breathing (1)
- ▢ b. Difficulty breathing at night (2)
- ▢ c. Cough, (3)
- ▢ d. Chest pain, (4)
- ▢ e. Dizziness, (5)
- ▢ f. Palpitations (racing, punding or fluttering heartbeat), (6)
- ▢ g. Swelling of the arms or legs (7)
- ▢ h. Loss of appetite, (8)
- ▢ i. Difficulty breathing when lying down (9)
- ▢ j. Vomiting, (10)
- ▢ k. Poor sleep (11)
- ▢ l. Others (please, specify).

IV) Do you follow medicine/drug recommendations suggested by your physician or other specialist?

- ○ a. Not at all (1)
- ○ b. Sometimes (2)
- ○ c. Usually (3)
- ○ d. Always (4)

V) If not always, why do you not follow medicine/drug recommendations in the question above (tick all that apply)

- ☐ a. Expensive to afford (1)
- ☐ b. Inconvenient to keep to frequency (2)
- ☐ c. Drugs not readily available even when you have the money. (3)
- ☐ d. The side effects are too many. (4)
- ☐ e. No one to remind and administer it sometimes (5)
- ☐ f. Fade up with constant medications. (6)
- ☐ g. Other (please, specify) (7)

SECTION C. PERCEPTION ON PHYSIOTHERAPIST-LED HEALTH PROMOTION

I) How helpful would it be to discuss the following with your physiotherapist?

	Not useful (1)	probably not useful (2)	very useful (3)
Exercise or physical activity (1)	☐	☐	☐
Diet (2)	☐	☐	☐
Weight management (3)	☐	☐	☐
Sleep (4)	☐	☐	☐
Stress issues (5)	☐	☐	☐

Alcohol intake (6)	☐	☐	☐
Giving up smoking (7)	☐	☐	☐
Managing your blood pressure, diabetes, or cholesterol (8)	☐	☐	☐
Medications (9)	☐	☐	☐
Othe therapies (e.g traditional medicine) (10)	☐	☐	☐
Other (Please, complete) (11)	☐	☐	☐

II) Which of the following applies to your recent experience of physiotherapy?

	Never (1)	Sometimes (2)	Always (3)
Has your physiotherapist ever made you uncomfortable about your condition? (1)	○	○	○
Can physiotherapists form good relationships with patients? (2)	○	○	○
Do you think the role of physiotherapists is limited to exercise prescription? (3)	○	○	○
Do you think that physiotherapists lack the skills to assist you with your condition? (4)	○	○	○
Has your physiotherapist discussed treatment goals and objectives with you? (5)	○	○	○

Does the physiotherapist praise and motivate you for reaching your goals? (6)	○	○	○
Do you think the physiotherapist workload is too much to allow them talk about your health? (7)	○	○	○
Do you tend to have similar views towards your condition as your physiotherapist? (8)	○	○	○

Section D. USEFULLNESS AND ACCEPTABILITY OF HEALTH PROMOTION PRACTICE

I) Which of these areas do you find useful in improving the management of your conditions? (tick all that applies).

	Not at all useful (1)	Moderately useful (3)	Very useful (4)
Increase general physical activity (1)	○	○	○
Increase specific exercise uptake (2)	○	○	○
Advice regarding eating more vegetables (3)	○	○	○
Advice for daily water intake (4)	○	○	○
Dietary advice to decrease fatty food intake (5)	○	○	○
Dietary advice to reduce excessive salt use (6)	○	○	○
Dietary advice to increase e fruits intake. (7)	○	○	○
Discussions on weight management. (8)	○	○	○
Explain the value of Body Mass Index as a measure of health (9)	○	○	○

Encouraging you to stop smoking. (10)	○	○	○
Education around normal sleeping patterns. (11)	○	○	○
Counsel to manage stress (12)	○	○	○
Counsel on alcohol consumption (13)	○	○	○
General advice on your main condition (	○	○	○

II) How happy are you to receive the following advice from a physiotherapist?

	Acceptable (1)	Undecided (3)	Unacceptable (5)
Increase general physical activity (1)	○	○	○
Increase specific exercise uptake (2)	○	○	○
Advice regarding eating more vegetables (3)	○	○	○
Advice for daily water intake (4)	○	○	○
Dietary advice to decrease fatty food intake (5)	○	○	○
Dietary advice to reduce excessive salt use (6)	○	○	○
Dietary advice to increase e fruits intake. (7)	○	○	○
Discussions on weight management. (8)	○	○	○
Explain the value of Body Mass Index as a measure of health (9)	○	○	○
Encouraging you to stop smoking. (10)	○	○	○

Education around normal sleeping patterns. (11)	○	○	○
Counsel to manage stress (12)	○	○	○
Counsel on alcohol consumption (13)	○	○	○
General advice on your main condition (14)	○	○	○

There is also an opportunity to take part in a second phase which will involve a more in-depth discussion about your experiences with physiotherapist and the services. If you are happy to participate, kindly provide your preferred contacts below and I will contact you in the next few weeks with further information to enable you take part in the research activity.

Name and Email

____

Phone number

____

We thank you for your time spent taking this survey. Your response has been recorded.

Kind regards,

Etienne Ngeh

S2: TOPIC GUIDE FOR THE SECOND PHASE

1. Thank you for agreeing to be interviewed for this research. Are you comfortable for me to have a recording of this discussion?
2. How are you doing health wise now?
3. When and why did you start attending physiotherapy?
4. From your previous experiences of Physiotherapy what type of management did you receive.

Prompt:

i. Did you receive any advice about changing any elements of your lifestyle i.e. smoking, sleeping, alcohol etc.?
ii. Are you currently maintaining any habit or lifestyle based on the advice you received from the physiotherapist?
iii. Explore specific components of lifestyle areas

5. In your own experience what are the greatest concerns about nature or type of physiotherapy services to enable you improve and have control over your health in this establishment?

Prompts:

i. Availability of what you need
ii. Cost of the service
iii. Nature of interaction with physiotherapists
iv. Delivery methods

6. Based on your past experiences with physiotherapists, do you find it useful in improving your health?

Prompts:

i. Are there some areas you find physiotherapists more useful than others?
ii. Are there some areas you think physiotherapists should improve on to better manage lifestyle related conditions?

7. In terms of qualifications and competence, what do you think of physiotherapists' ability to provide information, education, and resources to help people improve their health?
    i. Physical activity and exercise, alcohol use, weight management,
    ii. Specific conditions such hypertension and diabetes etc
8. How would you prefer physiotherapists to support you improve own condition? Considering things like exercise, diet, sleep, weight management, alcohol use etc
    i. Discussions with questions and answers
    ii. Written and printed materials
    iii. Regular meetings and education
9. Are you comfortable with physiotherapy approaches to discussing lifestyle conditions and health behavior change?

Thank you for your time and for providing responses to questions.

Supplementary file S3: Quantitative and qualitative results

1: Quantitative Results

1A: Participants perception of physiotherapist and physiotherapy services (n=146)

The majority of the respondents reported having a cordial interaction with physiotherapists (82.4%, n=120) and believe they can form good therapeutic relationships (79.5%, n=116). Physiotherapists were perceived as being competent in their role (60%, n=87). Respondents believed that the scope of physiotherapy practice is beyond exercise prescription (58.9%, n=89) and a proportion of respondents perceived that the workload of physiotherapists is always (18.6%, n=27) or sometimes (40.7%, n=59) too high to enable them to engage effectively in health education and promotional activities as reported in Table S1

Table S1: Participants experience and perceptions of PLPH (n=146)

**Which of the following applies to your recent experience of physiotherapy?**	Never		Sometimes		Always	
	%	n	%	n	%	n
Has your physiotherapist ever made you uncomfortable about your condition?	81.38%	118	13.79%	20	4.83%	7
Can physiotherapists form good relationships with patients?	2.05%	3	18.49%	27	79.45%	116
Do you think the role of physiotherapists is limited to exercise prescription?	58.90%	86	28.08%	41	13.01%	19
Do you think that physiotherapists lack the skills to assist you with your condition?	60.00%	87	20.00%	29	20.00%	29
Has your physiotherapist discussed treatment goals and objectives with you?	25.00%	36	27.08%	39	47.92%	69
Does the physiotherapist praise and motivate you for reaching your goals?	5.56%	8	27.78%	40	66.67%	96
Do you think the physiotherapist workload is too much to allow them talk about your health?	40.69%	59	40.69%	59	18.62%	27

1B: Perceived usefulness of PLHP interventions by pwCVDs (n=146)

Responses are summarised in Table S2; the majority of the respondents perceived PLHP interventions/advice to be very useful in improving their health and management of their conditions with mean score of 86.80% (n=124) across all components with 8.34% (n=12) considering it moderately useful and 4.79% (n=7) as not at all useful, Table S2. Higher proportions of perceived usefulness were reported for dietary advice on fruits (91.67%, n=132) and least was on advice or education to stop smoking (75. 35%, n=107).

Table S2: Perceived usefulness of elements of PLHP by pwCVDs (n=146)

**Which of these areas do you find useful in improving the management of your conditions? (tick all that applies)**	Not at all useful		Moderately useful		Very useful	
	%	n	%	m	%	n
Increase general physical activity	2.78%	4	9.72%	14	87.50%	126
Increase specific exercise uptake	2.07%	3	11.72%	17	86.21%	125
Advice regarding eating more vegetables	3.45%	5	7.59%	11	88.97%	129
Advice for daily water intake	3.50%	5	6.99%	10	89.51%	128
Dietary advice to decrease fatty food intake	5.59%	8	7.69%	11	86.71%	124
Dietary advice to reduce excessive salt use	1.40%	2	9.09%	13	89.51%	128
Dietary advice to increase e fruits intake.	3.47%	5	4.86%	7	91.67%	132
Discussions on weight management.	2.08%	3	11.11%	16	86.81%	125
Explain the value of Body Mass Index as a measure of health	4.23%	6	11.27%	16	84.51%	120
Encouraging you to stop smoking.	16.20%	23	8.45%	12	75.35%	107
Education around normal sleeping patterns.	3.50%	5	6.29%	9	90.21%	129
Counsel to manage stress	3.52%	5	9.86%	14	86.62%	123
Counsel on alcohol consumption	12.68%	18	7.04%	10	80.28%	114
General advice on your main condition	3.62%	5	5.07%	7	91.30%	126
Mean scores	4.79%	7	8.34%	12	86.80%	124

1C: Acceptability of PLHP interventions by respondents (n=146)

The majority of the respondents found PLHP advice/education acceptable across multiple components with a mean score of 94.65% (n=131). A small number of the respondents were undecided (3.54%, n=6) or found it unacceptable (2.34%, n=3) as reported in Figure S1. Increasing general physical activity was the highest component that pwCVDs were happy to receive advice (95.80%, n=137), and counsel on alcohol consumption was the component with the highest unacceptable respondents (4.8%, n=7).

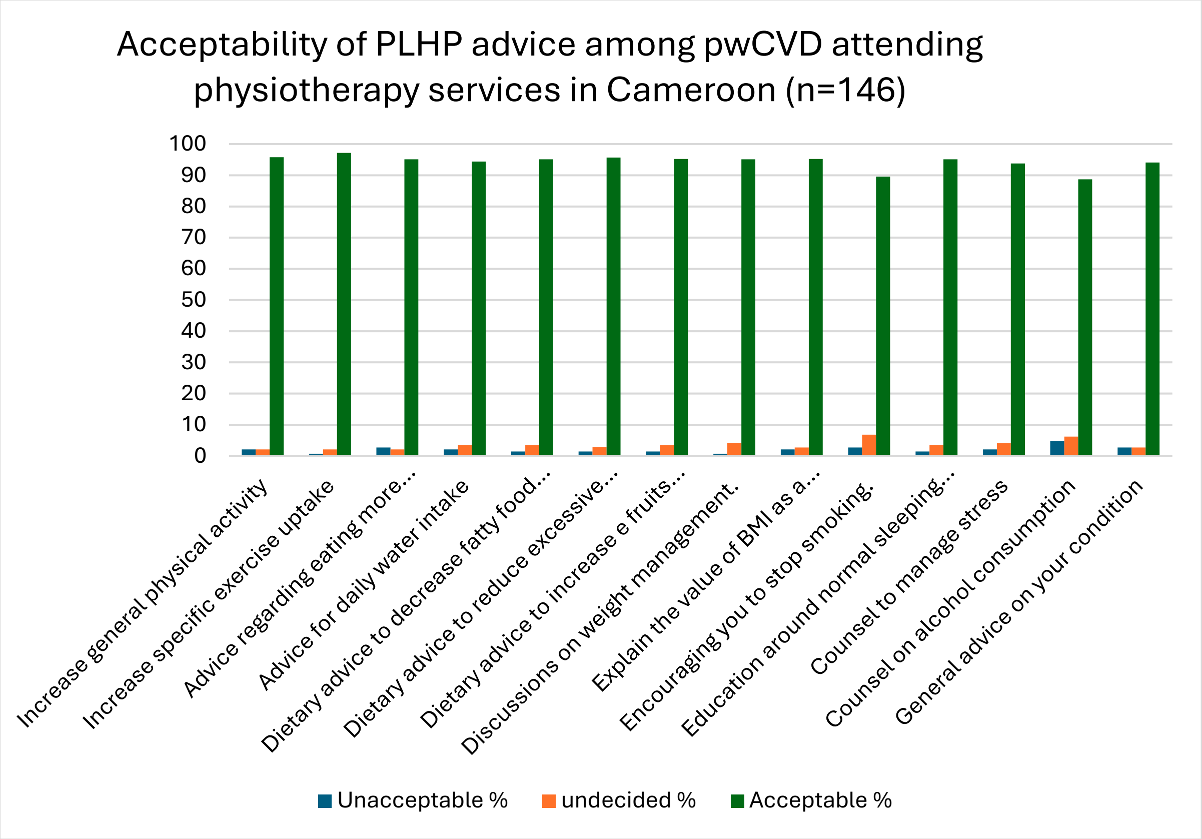

Figure S1: Components of PLHP that pwCVDs found acceptable (n=146)

2: QUALITATIVE RESULTS

	QUALITATIIVE FINDINGS IN BRIEF				
**Dimension**	**Higher Order Theme**	**Lower Order Theme**	**Files**	**References**	
Perceptions and experiences of pwCVDs on PLHP	Sources of information	Clinical referrals	5	12	"So, he referred me to follow up with a therapist, and that's what I'm doing presently" P10.
		Media	3	7	"They told me that I could not be treated. So, I did my own research and saw that the way out was physiotherapy and weight loss" P3.
	Reasons for attending physiotherapy	Primarily cardiovascular related	7	9	"When I suddenly had a stroke in 2013" P1, "I started physiotherapy because of insomnia and being overweight" P3.
		Cardiovascular secondary	6	8	" I was a victim of an accident. I was operated upon, and I was unable to move. I was bedridden. So, after the operation, the doctor advised that I could go for physiotherapy to help speed up the recovery. So, just like a week after the operation, I started physiotherapy" P7.
	Areas of PLHP practice	Exercise and Physical activity	13	46	"I did the therapy for about 6 weeks, and then they gave me a whole lot of exercise to go back home and do" P10.
		Diet and nutrition	8	16	"Yes, they have. I was advised to consume a lot of water. I was advised to consume a lot of fruits. Yes, they have" P7
		Alcohol use	9	15	"Even after I get better, I have been told that alcohol is detrimental to my health and wanting to stay healthy for the rest of my life, I won't go back to taking alcohol. It's an impediment" P11.
		Sleep	8	11	"For sleep and so on, I would think that I still need to have some in-depth knowledge and education" P5.
		components not covered	9	16	"What they do is just the exercise, but as concerns nutrition and other health issues. I think no one has ever told me about it" P11. "Yes. First, they should educate people even though they are not dietitians, but they should educate people on their diet" P9.
		Stress and psychological	8	10	"The way my physiotherapist talked, advice and handled my case, first of all, give me a kind of full psychological treatment" P1.
		Weight	4	9	"I need to watch my weight, because when the weight increases, the pain comes back" P9.
	Relationship with Physiotherapist	Positive	8	15	"Yes, if I were to make allusion the lady I am expecting to come and handle my situation, she is wonderful and her interaction. We encourage that kind of relationship if they can work on that in their workshops and seminars, that just the word coming from a physiotherapist to a patient, can even heal that patient psychological" P12.<br>"No, there are very interesting people, "he laughs" for my own sessions that I had were very interesting, very interesting. A lot of encouragement and you could see results" P3.
		Poor	3	6	"But others look a little bit, more of military kind of harsh to patients, some of them, but not all. Others are wonderful" P12.
	Practice of Physiot	Broaden the scope	5	9	"The physiotherapy unit is a very important unit. But again, I'll say they need to diversify their scope of training, it should be extended to other issues that affect health. P11

	herapists	of training			“So I think that physiotherapists as well, they need to be empowered in every other domain, because physiotherapy will also have to do with a change of lifestyle. It does with a change of lifestyle, so they should be empowered. They should know they should have a mastery of it, to help and educate the patients” P7.
		Professionalism	4	11	“The physiotherapist is being trained for what he does. The patient does not know what to do. So the physiotherapist should have that level of training that can take care of certain situations, because the patient is a novice, he doesn't know anything about physiotherapies. So the level of training should be improved” P11.
		Regulate training and practice	3	4	“So they really need to increase the number of people that are training in this area, because you go to some hospitals, you hardly find a physiotherapist. Then most of the time you have nursing aid assistant that have been trained informally in hospital settings, and they are handling this type of issues” P3.
	Factors affecting PLHP practice	cost	9	21	“Well for the cost. I won't say is quite affordable because someone who has to do physiotherapy, let's say for 2 months, 3 months and people go even beyond that. If you have to pay because, at the beginning, I had to come every day. So considering the standard of living here in our country, I think that the cost is not quite affordable for everyone” P7.<br>“Subvention for citizens: The government chooses to subsidize this issue, if they want the improvement of their citizens, it's not all about money the government rules citizens. It doesn't rule people. People pay taxes because of their health care” P11.
		Poor awareness and limited sensitization	9	18	“I think the national television station has to have good slots for education of this nature and the physiotherapist. And I think that a nation that consciously and formally facilitates and assists citizens to improve their health on things that they can do without a significant cost because they're doing exercises that they learn from the physiotherapists and so on, and they have the information, we be a healthier nation and the workforce will perform better” P3.<br>“So many people are really not educated here in Cameroon, they are really not aware or educated on the importance of a physiotherapist” P7.
		Limited training and employment in clinical practice	8	14	“Actually, I’m not aware of the capacity. I mean, how many people can be trained in this nation? Whatever the case, I think it is just a very small part. The ratio should be very low compared to the population” P1.<br>“As I’ve already said, most hospital don’t have that department, consequently, it's equally expensive, especially when it concerns the villagers, try to encourage even universities around to have a physiotherapy department, in their medical team. Try to encourage them” P1.
		limited home setting intervention	7	11	“That's why I would have loved for the physiotherapist to come for a home visit. To know the environment and even advise us on how to adapt to the environment. That's why I always like even home visits; I’m still striving for home visits” P5.

					"The government should assist, or NGOs should assist that is a physiotherapist produce a handout that can assist the patients while at home" P11.
		Limited policies related PLHP	7	8	"Still, it's not available all the times for people to be conscious about it, and it should be integrated into even our school programs, simple education, sensitization and communication to the population, part of public health, for example. Yes, let it be part of the public health program" P3.
		Lack clarity on health advice	4	7	"Yeah, because what I'm saying is that when you tell him to do some physical exercise, which by his very nature, he cannot do it, it means nothing. So it is good to tell somebody what he can do. I've had the experience where I'm told what to do, which I cannot do" P1.<br>"But it's different from here, because I have not had that opportunity of knowing what to do out of the service here" P10.
		Accessibility	3	4	"In fact, I would say that this service is an important service. Unfortunately, this service is only available in Bamenda as of now. Go to other divisions, other subdivisions; this department is lacking. Somebody has to leave, say, Nwa, Furawa, to come for an exercise" P12.
		Motivation	3	5	"What I think they can do to improve the service of physiotherapy is to pay them reasonable salaries that will enable others and make them want to come to practice. They should be many, there are few, they are not. There are not many as they are supposed to be like other doctors. They are just few so if there are many of them, it will help to reduce or cut down waiting time. So that is it" P6.<br>"Alone I cannot do it but when I come here, they are very specific. And I do not really like it alone because whenever I'm tired; I just get up and go. But here they say, no, 15 minutes, you still have 3 minutes and I make the effort" P13.
		Time	3	4	"They should be many, there are few, they are not. There are not many as they are supposed to be like other doctors. They are just few so if there are many of them, it will help to reduce or cut down waiting time. So that is it" P6.<br>"So, I think that the hospital should be equipped and probably recruit more people. Because of the number of patients coming into the hospital, the waiting time is quite long. So, I think they need to improve on that as well" P7.
		Workload	6	13	"So, I just felt that they have a lot of work, because the person that comes here is working elsewhere. He has a full-time work. So when he comes here, maybe he has to rush to his office, or has other patients to see, I just feel like they are not putting enough time" P4.<br>"I think everything boils down to time. Sometimes I feel that I don't have enough time, the workload at the level of the physiotherapist (long waiting time" P2
	Need for	specialist and	3	3	"So even though they're not dietitians, they can still play that role in talking to their patients about their diet". P9

	multidisciplinary approach to health	competence			
		Synergy	2	4	“But in the real treatment, I think that the specialists of this area are more competent if they want to take it as a disease to treat. But for the physiotherapists, I see him handling it as something resulting from his own specialty and something which his advice can equally help to check. But for treatment per say, I don't think so”. P1
	Confidence in Physiotherapists	Strong confidence	8	11	“The physiotherapist wants me to be well. So, I don't think that is any advice or restrictions that they can give me, which can be contrary to essence, I don't think so. What I got, help me and for me, what they do, I don't see anything to object” P4.<br>“My physiotherapist that is the way she talks. Her advice is very important, good that one is 100 %, she is confident doing what she's doing with me. I'm comfortable with what she's doing because I see that the practice that she gives me is workable with me” P6.
		Moderate confidence	6	10	“I cannot say that because what my physiotherapist is doing is helping me. So I cannot say that, she is trying her best”. P6<br>“I have no idea what they can bring in, but they are the ones who should advise me”. P8
	Perception of competence in PTs	Moderate	4	4	“I think that they should be empowered, it should be part and parcel of their everyday practice, I think so”. P7<br>“I think they are competent because I have not had any mal practices from them. They are competent, customer service, I can rate it 70 on 100 customer service. So, I think they are doing a good job” P10.
		Neutral	4	10	“When you go there you see somebody, you don't know his level of education, you don't know his competence. You are left in the hands of God and him. So, it is best for the administration to know and who to put in a particular position”. P11
		Poor	2	4	“Most of the time they are lacking, and they need somebody who should be overseeing them, who is actually very professional overseeing them, especially in the government settings” P3.
Usefulness and acceptability	components found to be more useful	No preference on education /advice	7	13	“What I know is that their advice, in general, is useful. Yes, I know that is useful, but to say, I can choose one for the other, no” P1.<br>“No, no, “Nodding in disagreement” they should talk about everything. They should talk of everything that will help me. That is it” P6.
		Diet and nutrition	4	4	“Yes dieting too, because if you do a lot of physical exercise without controlling your diet, then obviously you are not doing anything good” P9.
		physical treatment	3	5	“For me, their physical treatment, so when they put their patient on the bed and they are treating, that for me is a far more important, when you are in the hands of the physiotherapists, what he does is directly impact on the treatment” P1.
	Acceptability	Importance for the public	4	8	“So I say it is extremely, very, very important. This is why I'm saying that if it were possible, you should be able to open the department in most of the hospitals around” P2.<br>“They hold a transformational position in people's health” P9.

		Management of the condition	6	12	"When I suddenly had a stroke in 2013, it was necessary for me to get to physiotherapy for treatment. Well, I can say that was an important element in my treatment because they said it. Yes, I was heavily encouraged to do that. Let me add that it helped me a lot" P1.<br>"It helped me, and I saw that I followed the sequence of exercises, and they help me a lot. Yes, I believe that when you do the therapy and a therapist works with you the appropriate way, it will help you to improve on your health" P10.
Delivery methods	Preferred methods of PLHP	Individual approach	7	8	"By discussion, because when you discuss with someone, you have the possibility to ask questions where you have not been very clear about what has been said. So I think for me, it's by discussion" P7.<br>"But to in-patients, that individual approach can be okay, because if somebody is bedridden, and you have to treat the patient, you cannot take him to another place to join them, you cannot join them. So it is easy to talk to them individually" P1.
		Written or prints	6	12	"And besides that, there should be handouts. That is another level of improving, the physiotherapist prepares handouts. So, the physiotherapist should produce manuals" P11.<br>"You know, papers printed I will take and keep at home. You see, because I have sight problems as well. If it's maybe audio I can listen, but if it's written, I will take it and I will keep it. Well, you know people take papers, but they don't read" P1.
		no preference	6	7	"Anything that is going to enlighten me about my standard of living and educate me on how I'm supposed to live. No matter the means, I think I'm okay with that. If they are apps and whatever I don't mind" P7.
		Lecture format	3	5	"When the patients, especially the able patients, when they come together say 10 to 15 and so on, it is good that they are given a general lecture on what you're saying. That is on their meal, their sleep, their physical activities and so on. They should be given a general lecture, because talking to individuals, the language change from one person to the other" P10.<br>"So, they can always just talk to them in little groups as they come. They can sensitize them in the morning, before they start their work, it'll be important" P9.
		Regular talks or sessions	2	4	"If they have a session, once a month, for example, like my endocrinologist is in the military hospital, is a corner in the military hospital in Yaounde that's where I came from. He organizes what they say a "talk" every month with his patients and it's very helpful if there is something like this here, I will like that. I'll be coming" P13.<br>"But if I hear that, you people have organized a seminar or a come together to lecture people, I will still come. I'll be happy" P8.
		Audiovisual/media	2	2	"They can even do health talks on programs or health talk programs on radio, on television, why not even on flyers? For those will be able to read, they can read, but I think that physical, direct physical contact, visiting people in their groups, in the churches and all that will help or go a long way to make people

					understand what the role of the physiotherapist is in their community" P9.
		Home visits	2	2	"I would love that. But because I am having challenges, I think that doing it in a group might not be very comfortable working in a group. That's why I always like home visits; I'm still striving for home visits" P5.
		Use of apps	1	2	"By discussing and sending those apps. There are very educative apps I used to receive" P4.
		Patient support group (Forum for asynchronous communication)	1	1	"If someone has about 20 sessions, and then the physiotherapist has about 20 patients that he attends to. I think, to me, my own opinion, a forum should be made so that questions and answers daily. This is what I'm not going through. This will enhance the improvement of compliance". P11
